# Experience of Pleasure and Emotional Expression in Individuals with Schizotypal Personality Features

**DOI:** 10.1371/journal.pone.0034147

**Published:** 2012-05-16

**Authors:** Yan-fang Shi, Yi Wang, Xiao-yan Cao, Ya Wang, Yu-na Wang, Ji-gang Zong, Ting Xu, Vincent W. S. Tse, Xiao-lu Hsi, William S. Stone, Simon S. Y. Lui, Eric F. C. Cheung, Raymond C. K. Chan

**Affiliations:** 1 Neuropsychology and Applied Cognitive Neuroscience Laboratory, Key Laboratory of Mental Health, Institute of Psychology, Chinese Academy of Sciences, Beijing, China; 2 Graduate School, Chinese Academy of Sciences, Beijing, China; 3 Centre for Health Behavioural Research, School of Public Health and Primary Care, Chinese University of Hong Kong, Hong Kong Special Administrative Region, China; 4 Massachusetts Institute of Technology, Cambridge, Massachusetts, United States of America; 5 Department of Psychiatry, Beth Israel Deaconess Medical Center, Harvard Medical School, Boston, Massachusetts, United States of America; 6 Castle Peak Hospital, Hong Kong Special Administrative Region, China; Institute of Psychiatry at the Federal University of Rio de Janeiro, Brazil

## Abstract

Difficulties in feeling pleasure and expressing emotions are one of the key features of schizophrenia spectrum conditions, and are significant contributors to constricted interpersonal interactions. The current study examined the experience of pleasure and emotional expression in college students who demonstrated high and low levels of schizotypal personality disorder (SPD) traits on self-report questionnaires. One hundred and seventeen subjects with SPD traits and 116 comparison controls were recruited to participate. Cluster analyses conducted in the SPD group identified negative SPD and positive SPD subgroups. The negative SPD group exhibited deficient emotional expression and anticipatory pleasure, but showed intact consummatory pleasure. The positive SPD group reported significantly greater levels of anticipatory, consummatory and total pleasure compared to the control group. Both SPD groups reported significantly more problems in everyday memory and greater levels of depressive and anxiety-related symptoms.

## Introduction

Emotional impairments have been considered to be core features of schizophrenia [1], [2]. These impairments include both the reduced ability to experience pleasure (e.g., anhedonia) and to express emotion (e.g. affect) [3], [4], [5], [6]. However, most recent findings suggest that patients with schizophrenia often show a relatively intact ability to experience pleasure in response to positive laboratory stimuli [7], [8] regardless the impairment of hedonic capacity generated from clinical observations and self-reports studies [5], [6]. Empirical findings also suggest that ambivalent experiences of pleasure in schizophrenia may result from cognitive and motivational impairments [9], [10], [11], [12].

Deficits in the ability to experience pleasure in schizophrenia may also be divided into anticipatory (feeling of wanting) and consummatory (feeling of liking) dimensions [13]. Based on this framework, individuals with schizophrenia have been shown with intact consummatory pleasure but impaired anticipatory pleasure [13], [14], [15]. However, most of these studies were limited to patients with schizophrenia. Recent empirical findings from at-risk individuals for psychosis such as schizotypal personality disorder (SPD) traits or schizotypy [16], [17], [18], [19] also suggest that deficits in hedonic capacity may even be manifested in a subtle dysfunction even before the development of full-blown psychosis [20], [21], [22].

Studies of emotional processing often emphasize negative or positive aspects of SPD. Individuals with negative SPD (which is similar conceptually to negative schizophrenia symptoms), for example, often attend less than controls to their emotional states, while individuals with positive SPD (which is similar conceptually to positive schizophrenia symptoms), often attend more than controls to their emotional states [23]. Furthermore, depression and anxiety seem to be associated with more positive SPD than negative SPD [24], [25]. However, very little is known about the experience of pleasure and emotional expression in individuals with SPD.

Therefore, despite that a relatively substantial work on anhedonia has been done on schizophrenia spectrum disorders, most of them were limited to individuals who have developed a full-blown psychosis. Very little is known whether anhedonia has been demonstrated at-risk individuals with SPD features. The current study attempts to bridge the gap by exploring the experience of pleasure and emotional expression in individuals with SPD features. Given the heterogeneity of SPD, cluster analysis was used to identify different SPD subtypes. Cluster analysis is an empirical method used to group individuals based on their similarities/differences on selected variables, so that within-group differences are minimized and between-group differences are maximized [26], [27]. Previous studies have supported the existence of “negative SPD” and “positive SPD” clusters in non-clinical college students [28], [29].

We hypothesize that clusters of negative and positive SPD will be identified in a sample of college students who show elevated levels of SPD. We hypothesize further that individuals in the negative SPD cluster will show deficits in anticipatory pleasure, but will demonstrate intact consummatory pleasure and reduced emotional expressivity. By contrast, we expect that individuals with positive SPD will show a lesser extent of impairments as compared to the negative SPD in anticipatory and consummatory pleasure, and intact emotional expression.

## Methods

### Participants and procedure

Participants were screened out from a sample of 1039 college students aged 16–23 years old in four universities in Beijing, according to their scores on the Schizotypal Personality Questionnaire (SPQ) [30]. Subjects who scored in the top 10th percentile (SPQ score≥36) and the bottom 10th percentile (SPQ score≤10) of the screening sample served as the SPD and the comparison control groups, respectively. Consequently, 117 SPD (mean age = 18.44 years, standard deviation (SD = 0.83) and 116 control (mean age = 18.56 years, SD = 0.79) subjects were enrolled in the study. The groups did not differ significantly in age, years of education or gender (see Table 1).

**Table 1:** Characteristics of participants.

Variable	Individuals with SPD (*M±SD*)	Controls (*M±SD*)	Statistic^a^
Age	18.44±0.83	18.56±0.79	*t (231) = −1.17, n.s.*
Education (years)	12.23±0.61	12.21±0.81	*t (231) = 0.14, n.s*
Gender (male∶ female)	67 ∶ 50	75 ∶ 41	*X^2^(1, N = 233) = 1.34, n.s.*
SPQ	40.91±5.67	7.00±2.75	*t (231) = 57.98, p<0.001*
Cognitive-perceptual	18.64±4.74	3.87±1.98	*t (231) = 30.97, p<0.001*
Interpersonal	16.59±4.79	2.00±1.68	*t (231) = 30.96, p<0.001*
Disorganized	9.63±3.01	1.45±1.04	*t (231) = 27.68, p<0.001*

For abbreviation, SPQ: Schizotypal Personality Questionnaire.

a Two-tailed test.

### Measures

Questionnaires used in this study are described below. They were selected to identify subjects with high and low levels of SPD traits, and to assess the extent to which these subjects experience anticipatory and consummatory pleasure. Related factors, such their expression of emotion more generally, their memory for everyday events, and their levels of anxiety and depression were assessed as well.

#### Schizotypal traits

The Schizotypal Personality Questionnaire (SPQ) [30], [31] is a 74-item, true-false-response-format, self-report questionnaire. It is designed to assess 9 symptoms described in the *Diagnostic and Statistical Manual of Mental Disorders* (DSM-III-R) [32] criteria for schizotypal personality disorder (SPD). Although DSM SPD is not identical to Meehl's concept of schizotypy [18], [19], it serves as a useful approximation. Subjects who scored in the top 10% of the SPQ were classified as prone to develop or demonstrate SPD features. The SPQ shows good reliability and concurrent validity with other measures of schizotypal traits [30]. Its three-factor structure (i.e., *cognitive-perceptual*, *interpersonal* and *disorganized*) shows good internal consistency (i.e., Cronbach's α for *cognitive-perceptual* = 0.82, for *interpersona*l = 0.84, and for *disorganization* = 0.78) and cross cultural validation [33].

#### Experience of pleasure

The Temporal Experience of Pleasure Scale (TEPS) [15] is designed to measure both *anticipatory* (e.g. “I look forward to a lot of things in my life.”) and *consummatory* (e.g. “I enjoy taking a deep breath of fresh air when I walk outside.”) *pleasure*. The TEPS Chinese version contains 20 items with 6 Likert-format scales, and shows adequate overall reliability (Cronbach's α = 0.66) [34] and internal consistency for both anticipatory (Cronbach's α = 0.72) and consummatory (Cronbach's α = 0.78) items. Higher scores on this scale indicate greater degrees of pleasure.

#### Expression of emotion

The Emotional Expressivity Scale (EES) [35] is a 17-item, Likert-format questionnaire that assesses the ability to express emotions. Individuals rate themselves on a 6-point scale from 1 (Never) to 6 (Always) on how they express their emotions and feelings most of the time. A Chinese version of the questionnaire [36] showed high internal consistency overall (Cronbach's α = 0.82), and high internal consistency for ‘*suppression*’ (Cronbach's α = 0.82; e.g. “I keep my feelings to myself”) and ‘*expression*’ (Cronbach's α = 0.78; e.g. “I display my emotions to other people”) factors.

#### Everyday memory function

The Prospective and Retrospective Memory Questionnaire (PRMQ) assesses daily memory functioning [37]. It contains 16 items of prospective or retrospective memory-related activities in daily life, and shows good internal consistency (Cronbach's α = 0.88). Subjects rate the frequency and types of memory problems they encounter in daily life, with higher scores indicating greater problems. The current study adopted the Chinese version of PRMQ [38].

#### Depression and anxiety

These measures are intended to reflect general mental health. The Beck Depression Inventory (BDI) [39], [40] and the Trait Anxiety Inventory (T-AI) [41] were used to assess subject's experience of symptoms related to depression and anxiety. Both of these scales have 4-point-scale formats. Higher scores reflect more symptoms and/or higher levels of depressive symptoms and proneness to anxiety. The internal consistency of these scales is good (Cronbach's α for depression = 0.85, trait-anxiety = 0.86).

### Ethics statement

This study was approved by the Ethics Committee (i.e. an institutional review board) of the Institute of Psychology, Chinese Academy of Science, in Beijing. Written consent was required for participation and was obtained from all participants. Questionnaires were administered in a group format, and all participants received a course credit for their participation.

### Data analysis

SPSS 13.0 was used to analyze data statistically. Cluster analysis was performed with three factors of SPQ in the SPD group. Ward's hierarchical method with squared Euclidean distance was conducted first to explore the number of clusters, and then a K-means analysis was performed to confirm final cluster types [26]. One-way ANOVAs were used to assess group differences. Post hoc tests used the Least Square Differences (LSD) method to assess individual group differences in significant ANOVAs.

## Results

### Cluster analysis

Ward's hierarchical cluster analysis confirmed a two-cluster solution in the SPD group. Subsequent K-means cluster analysis further refined the clustering solution. The solution demonstrated one subgroup with prominent interpersonal difficulties that best corresponded to negative SPD, and a second group with prominent cognitive-perceptual problems that best corresponded to positive SPD. The two SPD groups and the comparison control group did not differ significantly in age, years of education, or gender ratio (see Table 2).

**Table 2:** Comparisons among groups.

	Group 1: Negative SPD (n = 55)	Group 2: Positive SPD (n = 62)	Group 3: Controls (n = 116)	F	*p*	*Least significant difference*
*Demographics*						
Age	18.39±0.85	18.48±0.82	18.56±0.79	0.87	0.420	1 = ^b^ 2 = ^b^ 3
Education (years)	12.15±0.51	12.29±0.68	12.21±0.81	0.57	0.569	1 = ^b^ 2 = ^b^ 3
Gender (male ∶ female)^a^	35 ∶ 20	32 ∶ 30	75 ∶ 41	3.11	0.212	1 = ^b^ 2 = ^b^ 3
*Schizotypal trait*						
SPQ: Cognitive-perceptual	16.11±4.99	20.89±3.14	3.87±1.98	642.24	**<0.001**	**2>** ^c^ **1>** ^c^ **3**
SPQ: Interpersonal	20.24±3.51	13.35±3.18	2.00±1.68	985.37	**<0.001**	**1>** ^c^ **2>** ^c^ **3**
SPQ: Disorganized	9.84±2.97	9.45±3.06	1.45±1.04	383.40	**<0.001**	**1**, **2>** ^c^ **3**
*Experience of pleasure*						
TEPS	77.36±14.63	88.84±9.64	80.64±14.29	12.14	**<0.001**	**2>** ^c^ **1**, **3**
Anticipatory	32.47±6.80	37.87±5.50	34.68±6.89	10.25	**<0.001**	**2>** ^d^ **3>** ^d^ **1**
Consummatory	41.53±9.07	47.32±6.13	42.28±8.50	9.86	**<0.001**	**2>** ^c^ **1**, **3**
*Expression of emotion*						
EES	54.05±12.73	60.35±12.89	63.31±9.64	12.43	**<0.001**	**2**, **3>** ^d^ **1**
Suppression	38.22±9.53	42.47±8.84	47.30±7.11	24.11	**<0.001**	**3>** ^d^ **2>** ^d^ **1**
Expression	15.84±4.87	17.89±5.25	16.01±4.47	3.75	**0.025**	**2>** ^e^ **1**, **3**
*Other measures*						
PRMQ	48.60±12.08	42.98±8.27	33.70±9.87	45.53	**<0.001**	**1>** ^d^ **2>** ^c^ **3**
BDI	13.51±10.18	10.03±6.35	1.28±2.01	89.63	**<0.001**	**1>** ^d^ **2>** ^c^ **3**
T-AI	50.89±8.69	46.52±8.64	34.41±6.31	106.65	**<0.001**	**1>** ^d^ **2>** ^c^ **3**

For abbreviations, SPQ: Schizotypal Personality Questionnaire; TEPS: Temporal Experience of Pleasure Scale; EES: Emotional Expressivity Scale; PRMQ: Prospective and Retrospective Memory Questionnaire; BDI: Beck Depression Inventory; TAI: Trait Anxiety Inventory.

a Gender.

### Experience of pleasure

Significant differences were obtained on the TEPS total score, *F*(2, 230) = 12.14, *p*<0.001, and on the *anticipatory*, *F*(2, 230) = 10.25, *p*<0.001 and *consummatory*, *F*(2, 230) = 9.86, *p*<0.001 scales. The negative SPD group showed the lowest experience of pleasure generally. Post hoc LSD tests showed that the negative SPD and the control groups did not differ significantly on the TEPS total score (p = 0.134), or on the *consummatory* pleasure score (p = 0.568). The negative SPD group did show significantly less *anticipatory* pleasure than controls (*p* = 0.040), however. In contrast, the positive SPD group demonstrated significantly higher levels than controls on all TEPS measures, including total score (*p*<0.001), *anticipatory pleasure* (*p* = 0.002) and *consummatory* pleasure (*p*<0.001). As to the comparison of the two SPD groups, the positive SPD group demonstrated significantly higher levels on all TEPS measures including total score (*p*<0.001), *anticipatory pleasure* (*p* = 0.002) and *consummatory* pleasure (*p*<0.001) than negative SPD group.

### Expression of emotion

Significant differences were obtained among three groups on EES total score, *F*(2, 230) = 12.43, *p*<0.001, on *suppression F*(2, 230) = 24.11, *p*<0.001, and on *expression*, *F*(2, 230) = 3.75, *p* = 0.025, with negative SPD showing the lowest levels of emotional expression. Post hoc LSD tests showed that negative SPD individuals reported lower scores on EES total (*p*<0.001) and *suppression* (*p*<0.001) than controls, but did not differ from them on levels of *expression* (*p* = 0.826). Although the positive SPD group and the control groups did not differ significantly on the EES total score (*p* = 0.099), the positive SPD group showed lower *suppression* (*p*<0.001) and higher *expression* (*p* = 0.013) than the control group. For the two SPD group comparison, the negative SPD group showed lower EES total score (p<0.001), lower *suppression* (*p*<0.001) and lower *expression* (*p* = 0.025) than positive SPD group.

### Memory for Everyday Events

The ANOVA for the PRMQ total score was significant, *F*(2, 230) = 45.53, *p*<0.001. Post hoc LSD tests showed that the negative SPD group reported more memory problems than the control group (p<0.001) and positive SPD group (p = 0.003), whereas controls demonstrated fewer problems than the other groups. The positive SPD group was intermediate between the other two groups.

### Depression and anxiety

Significant group differences were obtained on the BDI, *F*(2, 230) = 89.63, *p*<0.001, and on the T-AI, *F*(2, 230) = 106.65, *p*<0.001. Post hoc LSD tests demonstrated higher levels of depressive symptoms and anxiety proneness in both SPD groups, compared to the control group (ps<0.001). Moreover, the negative SPD group reported more depressive symptoms (*p* = 0.002) and higher anxiety proneness than the (*p* = 0.002) than the positive SPD group.

## Discussion

The current study showed that negative and positive SPD groups differ in their experience of pleasure and in their expression of emotion. Consistent with our hypothesis, the negative SPD group showed impaired anticipatory pleasure but relatively intact consummatory pleasure compared to controls. They also showed the least emotional expression among three groups, and reported the highest levels of problems with memory, depressive symptoms and trait-anxiety. The positive SPD group showed heightened pleasure experiences compared to controls, and similar total emotional expression, which provides partial support for our initial hypothesis. They also reported significantly greater problems with everyday memory, depressive symptoms and trait-anxiety than the control group, but significantly lower levels than the negative SPD group.

Findings of reduced anticipatory pleasure but intact consummatory pleasure in the negative SPD group are consistent with findings obtained in patients with schizophrenia [14]. Based on the view that deficits in anticipatory pleasure are related to deficits in motivation in schizophrenia [14], [15], our results raise the possibility that deficits in anticipatory pleasure may be related to deficits in motivation in negative SPD, too. Deficits in anticipatory pleasure in negative SPD may involve similar mechanisms to those that have been proposed for schizophrenia, such as difficulties in initiating approach behavior or in coupling the subjective experience of pleasure to the initiation or maintenance of goal-directed behaviors [42], [43].

In contrast to negative SPD, some of our findings concerning the experience of pleasure in positive SPD were unexpected. We hypothesized that the positive SPD group would show intact anticipatory and consummatory pleasure, and intact emotional expression. Interestingly, they showed a significantly heightened experience of overall, anticipatory and consummatory pleasure, compared to the control group. This may relate to previous findings of heightened emotionality and excessive attention to emotions in positive SPD [23], [44]. In one undergraduate sample, for example, higher levels of hallucinations (a positive symptom) were related to greater emotional arousal and fantasy-proneness [45].

The two SPD groups demonstrated distinct patterns of emotion expressivity with each other. The negative SPD group expressed the l lowest levels of emotions, while the positive SPD group did not differ from the controls in the total amount of emotional expression. However, after examining the scores of each factor of EES, findings showed that on the factor of *expression*, the negative SPD group scored lowest among the three groups, while the positive SPD group scored higher than controls. On the factor of *suppression*, both SPD groups displayed less emotional expressivity than the controls. Studies in SPD also show that during lab-based assessment of emotional processing, patients with schizophrenia demonstrated less emotional expression when watching sequentially-presented positive, neutral, and negative valence emotional stimuli. The decreased emotional expression in schizophrenia may be related to a decreased ability to amplify emotional expression. For example, Henry et al. [46] found that patients with schizophrenia demonstrated difficulty in amplifying their emotional expression behavior but did not show difficulty in suppressing it when they were asked to regulate their emotional expression when they watched amusing film clips. In another study, Henry et al. [47] also showed there was a similar decreased amplification of emotional expression, but not suppression in individuals with SPD. The current findings thus appear to be consistent with previous studies and demonstrate that negative SPD may have significant similarities to emotional expression with schizophrenia.

Findings from high risk groups suggest that high scores on anhedonia (negative feature) are related to high risk for schizophrenia [21], [22], [48] while high scorers on the Perceptual Aberration and Magical Ideation Scales (positive features) are at increased risk for future psychosis [49]. Our findings lend support on this view, although the connection between different high risk subtypes and schizophrenia remains unclear. The issue of whether individuals with prominent negative SPD features are more likely to develop schizophrenia, for example, or show poorer prognoses generally, requires additional research. Similarly, the issue of whether individuals with prominent positive SPD features are more likely to develop psychosis or to show better prognoses generally also requires additional study.

There are several limitations in our study. First, all of our participants were college students. Further study with more representative samples in the general population or in samples with clinically diagnosed SPD are needed to validate the current findings. Second, this study only used a series of self-reported scales to assess emotional processing and other dimensions of clinical and cognitive function. More objective experimental methods testing anticipatory and consummatory aspects of pleasure, emotional expression, and relationships between emotional processing and other relevant variables are needed, such as those involving specific anticipatory and motivational components of emotion experience [42], [50]. Future studies should also examine the underlying neural bases of emotional processing in SPD. Third, therapeutic intervention to SPD subjects would affect on their psychological performances. Although this study was not longitudinally-designed one, it would be possible that the individuals with SPD have been recommended to meet school counselors or psychologists previously. However, we did not officially count for this information. Nevertheless, we attempted to refer the SPD cases to the school counselors of their own colleges in order to take proactive action as much as possible. Last but not least, the current study did not administer a comprehensive battery or scales to either record socio-economic status of the participants or further differentiate subtyping of at-risk individuals such as schizoid personality who would potentially have the tendency of anhedonia. Future study should adopt a more systematic approach to get rid of any other personality subtypes that may confound the findings.

Nevertheless, the current findings demonstrate important differences between non-clinical individuals with negative and positive SPD traits. In particular, individuals with negative SPD showed more emotional and behavioral manifestations similar to patients with schizophrenia, while individuals with positive SPD tended to perform closer to controls in several, though not all, dimensions assessed.
